# Spoken Word Recognition across Language Boundary: ERP Evidence of Prosodic Transfer Driven by Pitch

**DOI:** 10.3390/brainsci13020202

**Published:** 2023-01-25

**Authors:** Juan Zhang, Yaxuan Meng, Chenggang Wu, Zhen Yuan

**Affiliations:** 1 Faculty of Education, University of Macau, Macau 999078, China; 2 Centre for Cognitive and Brain Sciences, University of Macau, Macau 999078, China; 3 School of Foreign Studies, Shanghai University of Finance and Economics, Shanghai 200433, China; 4 School of Education, Shanghai International Studies University, Shanghai 200083, China; 5 Institute of Linguistics, Shanghai International Studies University, Shanghai 200083, China; 6 Faculty of Health Sciences, University of Macau, Macau 999078, China

**Keywords:** Chinese EFL learner, pitch, prosodic transfer, spoken word recognition

## Abstract

Extensive research has explored the perception of English lexical stress by Chinese EFL learners and tried to unveil the underlying mechanism of the prosodic transfer from a native tonal language to a non-native stress language. However, the role of the pitch as the shared cue by lexical stress and lexical tone during the transfer remains controversial when the segmental cue (i.e., reduced vowel) is absent. By employing event-related potential (ERP) measurements, the current study aimed to further investigate the role of the pitch during the prosodic transfer from L1 lexical tone to L2 lexical stress and the underlying neural responses. Two groups of adult Chinese EFL learners were compared, as both Mandarin and Cantonese are tonal languages with different levels of complexity. The results showed that Cantonese speakers relied more than Mandarin speakers on pitch cues, not only in their processing of English lexical stress but also in word recognition. Our findings are consistent with the arguments of *Cue Weighting* and attest to the influence of native tonal language experience on second language acquisition. The results may have implications on pedagogical methods that pitch could be an important clue in second language teaching.

## 1. Introduction

Spoken word recognition plays a robust role in language processing, because it involves all aspects of the interface between the perception of low-level acoustic signals and the retrieval of high-level semantic information [1]. This process can be roughly divided into two stages: pre-lexical and lexical access [2,3]. Lexical access is mediated by the recognition of phonologically abstract pre-lexical unit forms, such as segmental and suprasegmental features. Only when it is based on the segmental and suprasegmental characteristics recognized at the pre-lexical level can lexical access be attempted [4].

The nature of spoken word recognition has been widely discussed in the literature, especially as it pertains to English, where lexical stress functions as the most prominent suprasegmental cue and could be further differentiated into three levels (primary, secondary, unstressed) [3,5,6,7,8]. English lexical stress manifests acoustically in multiple dimensions, including pitch (perceptual correlates of Fundamental frequency (F0)), duration, intensity, and vowel quality [9,10]. In comparison to primary or secondary stressed syllables (e.g., the first syllable in “decorative” or the first syllable in “disappear”), unstressed syllables (e.g., the first syllable in “prepare”) have relatively lower pitch and intensity, shorter duration, and vowels that can often be reduced to schwa [ə] [9]. Even though English is defined as a “stress” language, prior studies have found that English native speakers rely more on segmental properties than lexical stress cues to recognize spoken words due to the English language’s widespread tendency towards vowel reduction [11,12,13]. One such study, conducted by Cutler and Clifton, compared the effects of different types of mis-stressing (lexical stress shifts that did or did not involve vowel reduction) on word recognition [11]. Disyllabic nouns with either trochaic or iambic stress pattern were selected as stimuli and evenly divided into two groups. Half of the words in each group had vowels that were pronounced unreduced in both syllables and unreduced when stress shifted. The other half contained schwa in naturally pronounced weak syllables, which were pronounced unreduced when the stress pattern was manipulated. Although mis-stressing in words without schwa indeed hampered word recognition, the results showed that a segmental vowel cue played a more important role: reducing a full vowel or giving a schwa full weight made words harder to recognize. Similar results were obtained using a cross-modal priming paradigm. Minimal stress pairs, which share identical or near-identical segmental structures while containing distinct stress patterns without vowel reduction (e.g., *DIScount* vs. *disCOUNT*), were inserted in different auditory sentences as primes [14]. A visual word that was either semantically related or unrelated to the prime was shown on a screen during the presence of the auditory sentence. Meanwhile, participants were asked to judge whether this word was real or not. Consistently, the primes’ stress patterns did not affect the priming of target words, even though listeners could not rely on the absent segmental properties of vowels. These studies indicate that lexical stress does not play a role comparable to that of segmental information in English spoken word recognition. 

However, in languages where vowel reduction does not necessarily accompany lexical stress shift, such as Spanish, suprasegmental cues play a considerable role [15,16,17]. Soto-Faraco et al. explored the role of lexical stress in Spanish spoken word recognition using a cross-modal fragment priming (CMFP) approach. Word onset fragments of minimal stress pairs (the primes) were presented at the end of semantically neutral spoken sentences, accompanied by a visual letter string (the target word) at fragment offset. Participants were asked to judge whether or not those words were real. The results showed that both segmental and suprasegmental cues (i.e., lexical stress) were of great importance in the recognition of Spanish words [15]. In contrast, segmental mismatch was more effective in constraining the activation of competitors than suprasegmental mismatch in languages such as Dutch. Dutch occupies an intermediate state between English and Spanish in terms of the degree of vowel quality in lexical stress variation, meaning vowel reduction in Dutch is relatively voluntary [16].

### 1.1. Prosodic Transfer in Previous Studies

As previously established, when both segmental and suprasegmental cues are involved in English lexical stress variation, native speakers tend to rely on the former over the latter when identifying spoken words. Furthermore, the reliance on suprasegmental cues in L1 is found to influence how these cues are used when learning L2 stress languages [8,18]. For instance, Dutch EFL (English as a foreign language) learners who used lexical stress in their native language were found to outperform English native speakers in using lexical stress to judge English fragments, suggesting the influence of L1 background on L2 spoken word recognition [8]. Similarly, the lexical stress patterns in an artificial language were found to be learnt implicitly by English speakers after short exposure [18]. In contrast, speakers whose first language had fixed lexical stress placement showed difficulties in perceiving non-native lexical stress contrasts with unpredictable stress patterns [19,20]. Dupoux et al. used a sequence recall task involving short-term encodings of nonsense words with contrastive lexical stress to compare French and Spanish speakers’ abilities to perceive varied stress patterns. Unlike in Spanish, the lexical stress pattern in French is fixed, and the placement of stress is highly predictable as a functional marker of words and phrases’ final syllables [21]. In Dupoux’s study, French speakers performed worse than Spanish speakers in lexical stress contrast encoding, indicating a kind of “deafness” manifested by the influence of their native language [22]. Previous studies have attributed this influence to the carryover effect from L1 to L2 through prosodic transfer, namely the impact of L1 on L2 at the suprasegmental (e.g., lexical stress or tone) or phonetic/acoustic level (e.g., pitch or duration) [22,23,24,25].

More recent studies have demonstrated an increasing interest in investigating the influence of native tonal language on L2 stress language through prosodic transfer [25,26,27,28,29]. As a tonal language, both segmental and suprasegmental cues in Mandarin are lexically relevant. Completely different words may share the same segmental structure and differ only in tone [30]. There has been a great deal of debate surrounding how Mandarin EFL learners process English lexical stress and which cues are more heavily weighted. Some studies observe that while Mandarin and English have distinct dominant suprasegmental features, the acoustic correlates underlying the variation in these two features are partially shared, including F0, duration, and intensity [31,32,33,34,35]. Mandarin EFL learners rely on these shared acoustic cues to perceive L2 English lexical stress [28,29,36,37]. This phenomenon has been observed by Yu and Andruski, who compared the processing of English lexical stress between English native speakers and Mandarin EFL learners using three types of stimuli (real words, pseudowords, and hums) [29]. Hums were created by retaining a word’s original pitch and amplitude but removing all segmental information. The results showed that while both groups were able to discern English lexical stress, native speakers tended to exploit segmental cues (i.e., vowel quality), while Mandarin speakers relied primarily on pitch. When processing words that lacked segmental cues, such as hums, English speakers’ response times were even slower than when processing pseudowords. Similarly, by using nonsense disyllabic tokens with manipulated F0, intensity, and duration, Wang (2008) compared English speakers’ and Mandarin EFL learners’ reliance on distinct acoustic–phonetic cues. Wang’s results suggested that all three cues had significant effects on native English speakers’ stress perception, while only F0 had a decisive effect on that of Mandarin learners [36]. In both studies, tonal language background was observed to influence the way in which English stress was processed through prosodic transfer, and pitch plays a particularly robust role during this transfer.

However, other studies have found that like English native speakers, Mandarin learners might weigh vowel quality more heavily than other cues [26,38]. Using an identification task, Zhang and Francis asked Mandarin EFL learners to identify the grammatical category of presented auditory words (e.g., *DEsert* as noun vs. *deSERT* as verb). The results showed that Mandarin EFL learners relied more on vowel quality than on suprasegmental cues to process English stress [38]. Furthermore, no significant difference was found in the use of F0 and duration cues between Mandarin EFL learners and English native speakers. Mandarin speakers used vowel quality in combination with either F0 or duration to process English lexical stress. In addition, vowel quality and F0 were treated as combinational cues when discerning natural F0 contour but as independent cues when exposed to stimuli with relatively flat pitch contour. Zhang and Francis attributed this difference to the increased emphasis Mandarin learners placed on F0 direction as opposed to height in native language processing. Similar results were found by Chrabaszcz et al. [26]. In their study, the ability to identify the position of English lexical stress was compared among speakers of English, Russian, and Mandarin. The results indicated that compared to vowel quality, pitch only plays as a secondary cue by Mandarin speakers to perceive lexical stress, suggesting that Mandarin EFL learners make more use of vowel quality than pitch cue to process English lexical stress.

Due to the discrepancies found in previous studies, the current study aimed to further investigate the role of pitch in prosodic transfer from L1 tonal language to L2 stress language by comparing the utilization of pitch cues between Mandarin and Cantonese EFL learners. Specifically, the comparison in perceiving the contrast of English lexical stress and recognizing the spoken words was conducted when the cue of vowel reduction is absent.

### 1.2. The Current Study

Like Mandarin, Cantonese is a tonal language. Speakers use pitch cues to perceive tone variations in Cantonese [39,40,41]. Mandarin and Cantonese could be considered as two dialects of Chinese, with the most salient difference being the number of tones. There are six tones in Cantonese, including both contour tones and level tones [39], while there are only four contour tones in Mandarin [42]. Level tones are harder to distinguish than contour tones since they share the same pitch contour and differ only in pitch height [43]. To distinguish level tones, Cantonese speakers need to be more sensitive to relative F0 values and use this subtle height difference in their native language processing. Based on the previous studies discussed above, it was predicted that: (1) if pitch cue is dominantly used by Chinese EFL learners to process English lexical stress as a consequence of the prosodic transfer, then Cantonese EFL learners are expected to use more pitch information than Mandarin EFL learners to identify English lexical stress and recognize spoken words [44]; (2) if, instead, a cue such as the vowel quality is used by Chinese EFL learners to process English lexical stress, then the two groups are expected to perform equivalently in identifying English lexical stress and recognizing spoken words.

Two experiments were conducted to test the predictions. In Experiment 1, the utilization of pitch cues in the identification of English lexical stress was compared between Mandarin and Cantonese EFL learners. In Experiment 2, the use of pitch cues in recognizing English spoken words independently of segmental structure between the two groups was further investigated.

## 2. Experiment 1

In this experiment, the role of pitch in identifying English lexical stress was compared between Mandarin and Cantonese EFL learners. Using a prosodic identification task, our experiment aimed to investigate the role of pitch in perceiving English lexical stress. During the experiment, disyllabic words with trochaic stress patterns were changed to iambic words by shifting the F0 contour and vice versa. Event-related potentials (ERPs) were recorded while participants were asked to identify the words with artificial F0 contour.

In Experiment 1, we were mainly interested in the P200 component. P200 usually peaks before 300 ms after the onset of stimuli and reflects the processing of pitch information during spoken word recognition in such a manner that distinct F0 contours can be differentiated within the first syllables of words and indexed by the occurrence of P200 [45].

### 2.1. Methods

#### 2.1.1. Participants

Two groups of participants, including 16 native Mandarin speakers (10F/6M, mean age = 22.63 ± 2.68 years) and 16 native Cantonese speakers (10F/6M, mean age = 23.25 ± 2.74 years), were recruited from the University of Macau. The two groups were matched for age and gender (*p*s > 0.05). All the Mandarin speakers had been immersed in a Cantonese-speaking environment for less than one year, and the Cantonese speakers did not start to learn Mandarin until elementary school. According to the LexTALE test, both groups were classified as intermediate learners, who did not differ in English proficiency (Mandarin group: mean score = 60.19, SD = 4.34; Cantonese group: mean score = 60.56, SD = 4.94) [46]. Visions of all participants were normal or corrected-to-normal, and all participants were self-reported as right-handed.

Prior to the formal tests, all the procedures in both experiments were approved by the Institutional Review Board (IRB) of the University of the Macau. Informed and written consent forms were obtained from the participants. All assessments were carried out under the approved guidelines and regulations.

#### 2.1.2. Materials

**Figure 1:**
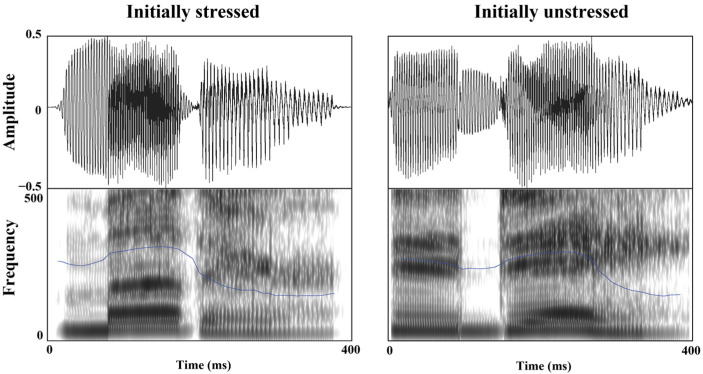
Waveforms (the upper row) and spectrograms (the lower row) of the initially stressed word “mother” and the initially unstressed word “today” in Experiment 1. The blue line represents the F0 contour of stress.

One hundred disyllabic words (fifty trochaic and fifty iambic words) with unreduced vowels in both syllables were selected from the English lexicon Project [47] and recorded by one female English native speaker in a sound-attenuated room. The word frequency and length (SUBLEX-US) were matched between trochaic and iambic disyllabic words (*ps* > 0.05) (Table 1) [48]. The familiarities of the 100 words were evaluated individually by 20 Chinese EFL learners who did not participate in the experiment using a 7-point scale (1–7: from unfamiliar to familiar), and were matched between the 2 stress categories, *t* (98) =1.75, *p* ˃ 0.05. To eliminate the influence of duration and intensity, F0 contours were extracted from the trochaic word *MOther* and iambic word *toDAY* using PRAAT [45]. Based on the extracted F0 contours, each experimental stimulus was re-synthesized into two versions, one with the trochaic F0 contour from *MOther* and the other with the iambic F0 contour form *toDAY* [34] (Figure 1).

#### 2.1.3. Procedure

Using E-prime 2.0, the experimental materials were presented to the participants. During the formal experiment, participants were asked to sit in front of a computer screen. In each trial, a fixation cross was first presented in the centre of the screen for 500 ms, followed by the auditory stimulus through the headphone with the cross fixed on the screen. After the presentation of the auditory stimulus, the cross was replaced by a blank screen, and the participants judged whether the prosodic stress pattern of the heard word was correct or not by pressing the Serial Response Box (SRBox, Psychology Software Tools, Inc, Pittsburgh, PA, USA). Participants were asked to respond as quickly and accurately as possible. All 200 stimuli were presented to participants through 2 blocks. One block contained fifty correctly stressed words (half trochaic and half iambic) and fifty incorrectly stressed words (half trochaic and half iambic). The other block contained 50 correctly stressed words that were pronounced incorrectly in the former block and the incorrect version of words that were correctly stressed in this block. One block only contained either the correct or incorrect stress pattern of a word. Words in each block and the two blocks were randomly presented to the participants. Any responses that were given beyond 2000 ms or before the offset of stimuli were not recorded.

#### 2.1.4. EEG Recordings and Analysis

Electroencephalograph (EEG) data were recorded from the EGI 128 electrode Hydro Cel Geodesic Sensor Net, which was linked to Net Station 4.5.6 software sampled at 1000 Hz. The impedances of all the electrodes were reduced to below 50 kΩ before each block. The signal was first filtered with a 0.1–30 Hz band-pass and then segmented into 700 ms epochs (100 ms before the presentation of the target and 600 ms after the presentation of the target). An artifact detection criterion was set to 140 μV for eye blink segments, 140 μV for eye blinks, 55 μV for eye movements, and 200 μV for bad channels. A channel was marked as bad if more than 20% of the segments met the above-mentioned criteria. Meanwhile, segments were discarded if there were more than 10 bad channels, with an eye blink or with an eye movement. Bad channels were interpolated with averaged data from the remaining good channels using spherical splines, and the ERP segments were then averaged for each participant. A participant with an acceptance rate lower than 70% was excluded. There is no significant difference between the two groups in the mean number of retained epochs (Mandarin: 39.98 ± 2.52; Cantonese: 40.52 ± 2.77). All waveforms were then re-referenced to an average reference, and a 100 ms pre-stimulus baseline correction was applied.

**Figure 2:**
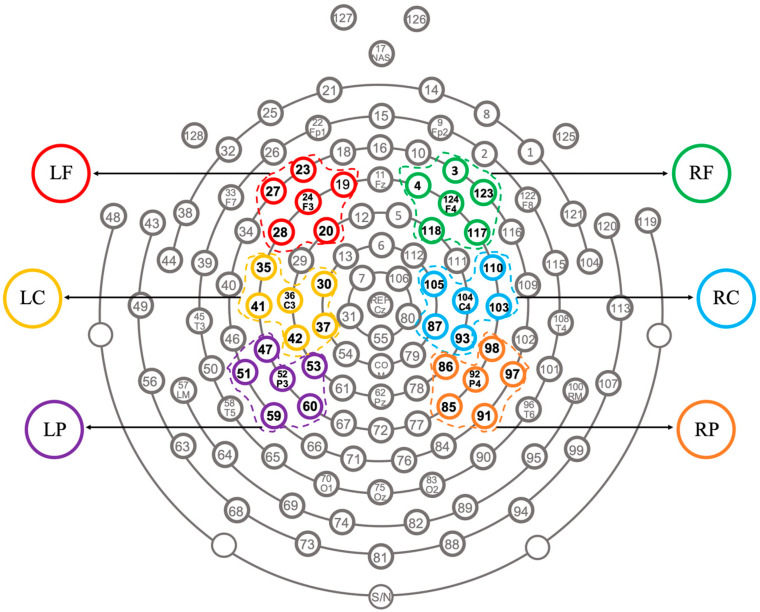
Illustration of the six electrode clusters used for the EEG analysis.

From 0 to 600 ms after the onset of the visual target, the mean amplitude of the ERP waveforms for each condition was calculated over each successive 50-ms time window. Within each time window, repeated measures ANOVA was conducted with *Group* (Cantonese vs. Mandarin) as the between-subject variable and *Stress Pattern* (Trochaic vs. Iambic), *Correctness* (Correct vs. Incorrect), and *Region* as the within-subject variables. For the variable *Region*, electrodes were grouped into six regions as follows (see Figure 2): left frontal (LF: 19, 20, 23, 24, 27, 28), right frontal (RF: 3, 4, 117, 118, 123, 124), left central (LC: 30, 35, 36, 37, 41, 42), right central (RC: 87, 93, 103, 104, 105, 110), left parietal (LP: 47, 51, 52, 53, 59, 60), and right parietal (RP: 85, 86, 91, 92, 97, 98). ERP amplitudes of the electrodes in each cluster/region were averaged, and then the obtained mean ERP value for each condition was taken into further analysis. To avoid the likelihood of a Type I error due to the large number of comparisons, only those effects which reached significance in two or more successive time windows were identified as real effects. Greenhouse–Geisser correction was used to test the violation of sphericity.

### 2.2. Results

#### 2.2.1. Behavioural Results

Repeated measures ANOVA with *Group* as the between-subject factor, *Correctness* and *Stress Pattern* as within-subject factors was conducted. Response time (RT) and accuracy data for different conditions are presented in Table 2. For the RT, the main effect of *Group* was significant that Cantonese speakers responded faster than Mandarin speakers across conditions (*F* (1, 30) = 9.36, *p* < 0.05). The main effect of *Correctness* was significant (*F* (1, 30) = 11.11, *p* < 0.05) that correct words were processed faster than incorrect words. Meanwhile, the main effect of *Stress Pattern* was also significant (*F* (1, 30) = 21.17, *p* < 0.001) that trochaic words were processed faster than words with iambic pitch contour. No significant interaction was found in RT analyses.

For accuracy rate, the main effects of *Correctness* and *Stress Pattern* were both significant (*F*_1_ (1, 30) = 68.84, *p*_1_ < 0.001; *F*_2_ (1, 30) = 174.17, *p*_2_ < 0.001). However, the interactions between *Correctness* and *Stress Pattern*, *Group* and *Correctness* were also significant (*F*_1_ (1, 30) = 47.67, *p*_1_ < 0.001; *F*_2_ (1, 30) = 7.38, *p*_2_ < 0.05). Bonferroni post-hoc analyses showed that for both correct and incorrect words, the accuracies of words with a trochaic pitch contour were higher than those with an iambic contour (*F*_1_ (1, 30) = 14.04, *p*_1_ < 0.05; *F*_2_ (1, 30) = 162.47, *p*_2_ < 0.001). In addition, for words with either a trochaic or iambic pattern, the accuracy of correct words was higher than incorrect ones (*F*_1_ (1, 30) = 21.04, *p*_1_ < 0.001; *F*_2_ (1, 30) = 89.91, *p*_2_ < 0.001). For the interaction between *Group* and *Correctness*, the results showed that for both groups, the accuracies in processing correct words were higher than incorrect ones (*ps* < 0.05). Meanwhile, the accuracy was higher in the Cantonese group than in the Mandarin group when processing correct words (*F* (1, 30) = 8.17, *p* < 0.05).

#### 2.2.2. ERP Results

**Figure 3:**
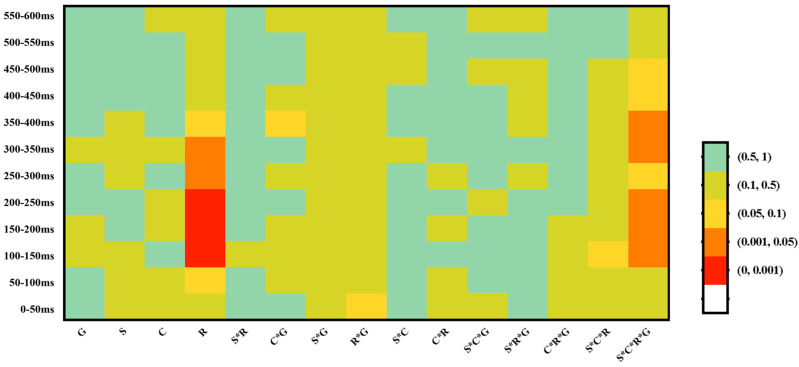
Repeated measures ANOVA was conducted with *Group* (Mandarin vs. Cantonese) as the between-subject variable, *Correctness* (Correct vs. Incorrect), *Stress Pattern* (Trochaic vs. Iambic), and *Region* as the within-subject variables. *P*-values of these analyses are colour coded. The mean signal violation was calculated over each successive 50 ms time window from 0 to 600 ms after the onset of the stimuli in Experiment 1. *G,* Group; *C*, Correctness; *S*, Stress Pattern; *R*, Region.

**Figure 4:**
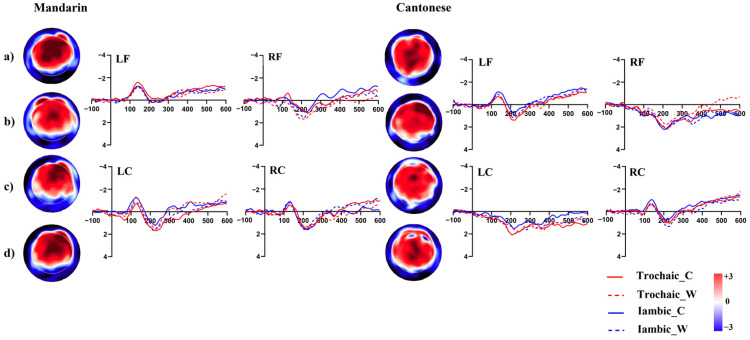
Grand average topographies and ERPs of the P200 (100–250 ms) component across conditions in Experiment 1 (**a**): correct trochaic words; (**b**): incorrect trochaic words; (**c**): correct iambic words; (**d**): incorrect iambic words.

Results of the 12 successive 50 ms time-windows analyses are summarized in Figure 3. Based on the exploratory analysis, the P200-like positivity was tested in a 100–250 ms. Grand average ERP waveforms are illustrated in Figure 4. 

In the time window from 100 to 250 ms, a four-way interaction involving *Correctness*, *Stress Pattern*, *Region*, and *Group* was significant, *F* (5, 150) = 3.03, *p* < 0.05. Simple effect analyses were conducted to further investigate significant three-way interactions at different levels of the fourth variable. For the trochaic word condition, significant three-way interaction was found among *Correctness, Region,* and *Group*, *F* (5, 150) = 4.06, *p* < 0.05. Further analysis of this interaction showed that in the processing of correct words, the amplitude of P200 was more positive in the Cantonese group than in the Mandarin group within the left frontal region (*F* (1, 30) = 5.84, *p* < 0.05). For the incorrect word condition, no significant interaction was found. In addition, significant three-way interactions among *Correctness*, *Stress Pattern*, and *Group* were found at the left central region, *F* (1, 30) = 8.73, *p* < 0.01. The amplitude of P200 in the processing of correct words with a trochaic pattern was more positive than those with an iambic pattern in both groups within this region (*F*_1_ (1, 30) = 5.86, *p*_1_ < 0.05; *F*_2_ (1, 30) = 5.03, *p*_2_ < 0.05).

### 2.3. Discussion

Experiment 1 was conducted to compare the potential differences in how Mandarin and Cantonese EFL learners used pitch information to process English lexical stress. Both groups responded faster and more accurately to correct words than to incorrect words, as well as to words with a trochaic stress pattern than to those with an iambic one, indicating that both groups could use pitch information to perceive English lexical stress. With regards to how the two groups compared, the Cantonese group responded significantly faster than the Mandarin group across conditions, and the accuracy in the Cantonese group was higher than the Mandarin group in the processing of correct words, which suggested that the Cantonese group was more sensitive to the variation in pitch cue during the perception. In addition, a significant group difference was also found in the ERP analysis: in the processing of correct words with trochaic pattern, the amplitude of P200 was more positive in the Cantonese group than in the Mandarin group within the left frontal region.

As previously discussed, P200 can reflect the perceptual processing of acoustic cues, whose amplitude is related to the F0 contours which underlie the variations in lexical stress [45,49]. Furthermore, results of recent studies have shown that auditory training influence the amplitude of P200 [50,51]. For instance, Marie et al. found that the P200 effect was larger for musicians than for non-musicians, suggesting that extended musical training could influence the amplitudes of P200 [50]. In line with these studies, our study found that the amplitude of P200 was more positive in the Cantonese group than in the Mandarin group in the processing of correct words, which could be attributed to the result of intensive pitch training due to the effect of language experience. Compared to Mandarin, Cantonese has more complex tones and speakers rely more on pitch cues to differentiate level tones [39]. The reliance on pitch cues in daily life could be considered as insensitive pitch training, which resulted in the improvement of the Cantonese group’s perception of English lexical stress and induced neural changes as reflected by the modulations of the amplitude of P200.

Both Behavioural and ERP results were consistent with our hypothesis that if pitch cue is dominantly used by Chinese EFL learners to process English lexical stress as a consequence of the prosodic transfer, then Cantonese EFL learners should use more pitch information than Mandarin EFL learners to identify English lexical stress. Along with the studies by Wang (2008) and Yu and Andruski (2010) [29,36], our results in Experiment 1 supported the view that Chinese EFL learners rely on the shared acoustic cues by lexical tone and the lexical stress to perceive English lexical stress [28,29,36,37]. In other words, the transfer from L1 lexical tone to L2 lexical stress occurs at the phonetic level, and pitch plays a robust role in the transfer. Those who use more pitch cues in their native language processing will keep using such cues in the perception of second language. Therefore, Cantonese learners outperformed their Mandarin counterparts in perceiving English lexical stress and were found to use more pitch cue in the perception.

## 3. Experiment 2

In this experiment, the role of pitch in English spoken word recognition was further compared between Mandarin and Cantonese EFL learners. Using a cross-modal fragment priming task, we investigated whether the pitch cue on its own, independent of any segmental structure, was sufficient for listeners to recognize English spoken words. Auditory word fragments (e.g., *mu-*) extracted from initially stressed or unstressed words (e.g., *MUsic* or *muSEum*) were presented. Participants were asked to judge whether the subsequent visual word was real or not. The N400 component, which usually peaks at about 400 ms after the onset of stimuli, was investigated. N400 reflects cognitive processing in word recognition and semantic integration. Its amplitude is sensitive to the repetition of phonological or orthographic information, decreasing when repeated information facilitates semantic retrieval [52,53,54].

### 3.1. Method

#### 3.1.1. Participants

Two groups of participants (Mandarin: 10F/6M, mean age = 22.69 ± 2.63 years; Cantonese: 9F/7M, mean age = 24.00 ± 3.46 years) who did not participate in Experiment 1 were recruited for Experiment 2. The two groups were matched for age and gender (*p*s > 0.05). According to the LexTALE test, the participants in both groups were intermediate EFL learners and did not differ in terms of English proficiency (Mandarin group: mean score = 60.38, SD = 4.08; Cantonese group: mean score = 61.06, SD = 4.20) [46].

#### 3.1.2. Materials

Based on a previous study, 20 disyllabic word pairs were adopted as experimental materials [8]. Specifically, each word pair overlapped segmentally in the first syllable but had different stress positions, either on the first or second syllable, without vowel reduction. All the disyllabic words were recorded by one female English native speaker. The initial syllables of the 40 words were extracted as auditory fragments using Cool Edit (Syntrillium Software Corp., Phoenix, AZ, USA). In total, 40 auditory fragments were extracted as primes. The original disyllabic words were presented as visual target words. Each target word was presented four times to guarantee the number of trials. In addition, there were 160 filler words, consisting of a set of 80 words that matched the auditory fragments suprasegmentally but not segmentally, and another set of 80 words that mismatched the fragments both segmentally and suprasegmentally. The familiarity of these target and filler words was assessed by an independent group of Chinese EFL learners that did not participate in Experiment 2 using a 7-point scale. The word length, frequency, and familiarity were matched across conditions (*p*s > 0.05) (Table 3). In addition, 320 pseudowords (matching the numbers of real words) that matched the real words in word length were created using the English Lexicon Project (ELP) [47].

**Figure 5:**
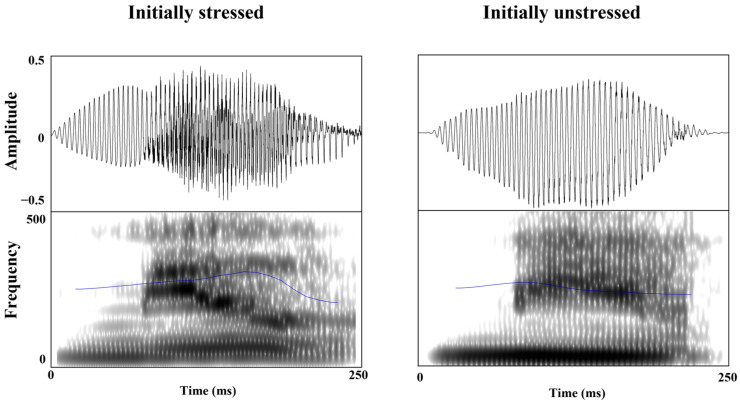
Waveforms (the upper row) and spectrograms (the lower row) of the two resynthesized versions of the syllable *mu-* taken from the word *music/museum* in Experiment 2. The blue line represents the F0 contour of stress.

The duration of the 40 auditory fragments, the time point of onset and offset, and the value of the highest pitch were extracted and calculated using PRAAT. In stressed syllables, the average F0 value of a voicing onset is 251.18 Hz at 43.78 ms, a pitch maximum is 308.01 Hz at 170.76 ms, and a voice offset at 238.99 ms with 269.64 Hz. Compared to stressed syllables, unstressed syllables were characterized by a voice onset at 37.78 ms with 246.12 Hz, a pitch maximum at 83.79 ms with 275.16 Hz, and a voice offset at 214.99 ms with 230.19 Hz. Different parameters were interpolated to construct typical stressed or unstressed templates (Figure 5). The original auditory fragments were standardized on the basis of the trochaic and iambic templates with PRAAT by keeping the duration and amplitude of the syllable constant.

#### 3.1.3. Procedure

During the experiment, participants were asked to sit in front of a computer screen, and in each trial, an auditory fragment was presented to participants through headphones after a fixation cross in the centre of the screen for 500 ms; the presentation time of a given auditory fragment varied based on the duration of the fragment. Immediately after the auditory fragment, a visual word was presented on the screen. The participants were instructed to decide whether the word was real or not as quickly as possible using the SRBox. Each fragment was presented 16 times for a total of 640 prime–target pairs. Any response given later than 2000 ms after the onset of the visual stimuli or before the offset of the stimuli was not recorded. The interval between the two trials was fixed to 1000 ms. The order of blocks was counterbalanced across participants. 

#### 3.1.4. EEG Recordings and Analysis

The recordings and analysis in Experiment 2 were the same as those in Experiment 1. The average number of epochs included did not differ between the two groups (Mandarin: 31.58 ± 2.27; Cantonese: 31.71 ± 2.33).

### 3.2. Results

#### Behavioural Results

Repeated measures ANOVA with *Segmental congruency* and *Pitch congruency* as within-group variables and *Group* as the between-group variable was conducted (see Table 4). For the RT, the main effect of *Group* was significant that Cantonese speakers responded faster than Mandarin speakers across conditions (*F* (1, 30) = 19.38, *p* < 0.001). The main effect of *Segmental congruency* was also significant (*F* (1, 30) = 60.37, *p* < 0.05) that the processing of segmental matched targets was faster than segmental mismatched ones. 

For the accuracy, the main effect of *Segmental congruency* was significant (*F* (1, 30) = 38.72, *p* < 0.001) in that the accuracy of segmental-matched targets was higher than mismatched ones. No other significant main effect or interaction was found.

**Figure 6:**
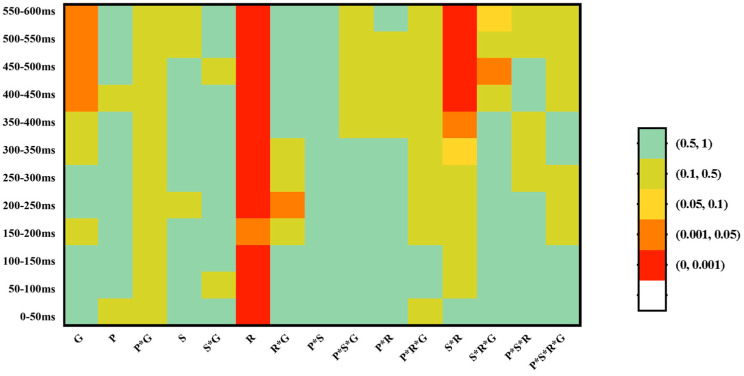
Repeated measures ANOVA was conducted with *Group* (Mandarin vs. Cantonese) as between-subject variable, *Segmental congruency* (congruent vs. incongruent), *Pitch Congruency* (congruent vs. incongruent), and *Region* as the within-subject variables. *P*-values of these analyses are color coded. The mean signal violation was calculated over each successive 50ms time window from 0 to 600ms after the onset of the stimuli in Experiment 2. *G*, Group; *P*, Pitch Congruency; *S*, Segmental congruency; *R*, Region.

**Figure 7:**
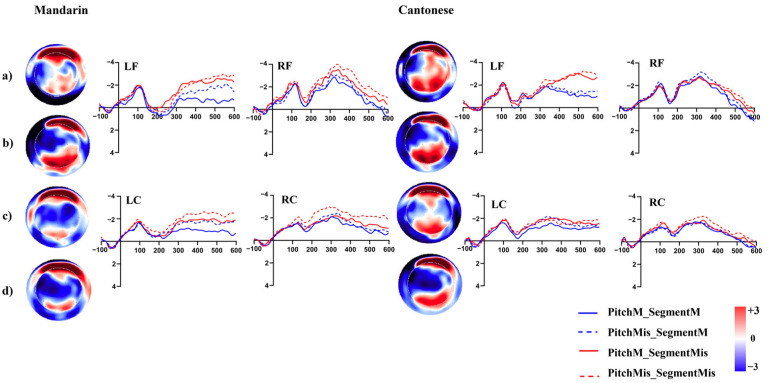
Grand average topographies and ERPs of the N400 (400–600ms) component across conditions in Experiment 2. (**a**): target that matched with the prime segmentally and in pitch contour; (**b**): target that matched with the prime in pitch contour but not segmentally; (**c**): target that matched with the prime segmentally but not in pitch contour; (**d**): target that mis-matched with the prime segmentally and in pitch contour.

Successive time-windows analyses in Experiment 2 are summarized in Figure 6. Based on the exploratory analysis, the N400-like negativity was analysed in the 400–600 ms time window. Grand average ERP waveforms are illustrated in Figure 7. Repeated measure analyses of variance (ANOVAs) were conducted for the mean amplitudes of N400, with *Segmental congruency*, *Pitch congruency*, and *Region* as the within-subject factors and *Group* as the between-group factor. Greenhouse–Geisser correction was used to test the violation of sphericity.

The N400 results showed that the main effect of *Group* was that the amplitude of N400 observed among the Cantonese group was less negative than that of the Mandarin group, *F* (1, 30) = 4.528, *p <* 0.05. The main effect of *Region* was significant (*F* (1, 30) = 8.69, *p <* 0.001). The interaction between *Segmental congruency* and *Region* was also significant (*F* (5, 150) = 9.71, *p <* 0.001). Post-hoc analyses were further conducted and the results indicated that the amplitude of N400 was more negative in the processing of segmental-mismatched words than matched ones within the left frontal region (*F* (1, 30) = 19.47, *p <* 0.001). Meanwhile, the left frontal region was more negatively activated than the right frontal and central regions (*F*_1_ (1, 30) = 9.21, *p*_1_ *<* 0.05; *F*_2_ (1, 30) = 34.76, *p*_2_ *<* 0.001). 

### 3.3. Discussion

Experiment 2 compared whether Mandarin and Cantonese EFL learners used pitch information differently when recognizing English spoken words. Behavioural results such as accuracy and response time were consistent within both groups in that the processing segmental-matched targets were faster and more accurate than mismatched ones. A notable difference between the Mandarin and Cantonese groups was found only in response times: the Cantonese group responded significantly faster than Mandarin speakers in subsequent visual word recognition across all conditions.

The N400 component was examined to study how participants’ use of pitch information in lexical access influenced semantic retrieval. As previously discussed, the N400 component could reflect cognitive processes related to word recognition and semantic integration. In Chinese, the role of pitch in constraining spoken word recognition is comparable to that of segmental information, and both segmental and pitch violations induce negative N400 [55,56]. Therefore, the difference in N400 amplitude between the Mandarin and Cantonese groups (the Cantonese group had a smaller N400 response) could be interpreted as reflecting top-down processing where language background influences the recognition of speech [57]. In line with the results in Experiment 1, the results suggested that compared to the Mandarin group, Cantonese speakers rely more on pitch information in their native language processing and, influenced by their native linguistic background, the Cantonese group recognizes L2 spoken words with less effort, manifested in smaller N400 amplitude [58].

## 4. General Discussion

The two ERP experiments were conducted to compare the role of pitch between Mandarin and Cantonese EFL learners in the processing of English lexical stress and the recognition of English words. Our results indicated that while both groups used pitch information to identify English lexical stress, the Cantonese group relied more on pitch information than the Mandarin group. Furthermore, we observed that a language-specific reliance on pitch cue affects the recognition of English spoken words. The more speakers use pitch cues in L1, the more they tend to rely on pitch information in L2 processing.

We have interpreted these results in light of the associations between acoustic cues and spoken word recognition. As previously discussed, variations in Chinese lexical tone are mainly achieved by changing acoustic cues such as pitch, duration, and intensity. These acoustic cues are also dominant constituents in English lexical stress shift [34,59]. Based on the two languages’ shared underlying acoustic cues, Chinese EFL learners are able to rely on lexical stress in English spoken word recognition through a prosodic transfer at the phonetic level. Meanwhile, native linguistic background influences speakers’ bias toward the extraction and utilization of acoustic–phonetic cues in word recognition [60,61,62]. Chinese EFL learners are accustomed to pitch playing a dominant role in the variation of lexical tone: for example, determining the lexical tone of a Chinese syllable based on the pitch information of its main vowel [63,64]. It follows that the utilization of pitch information in L1 could influence how such information is used in L2 spoken word recognition [22,23]. Cantonese speakers, who rely more on the pitch than Mandarin speakers, especially in relation to pitch height [39,44], tend to use more pitch information in L2 spoken word recognition. Our results are consistent with the arguments of *Cue Weighting* [60,61,62]. According to these arguments, multi-dimensional acoustic–phonetic cues are perceived and weighted in speech, and the weights of these cues differ across languages. Speakers learn phonetic categories as they shift attention to various acoustic cues during their native phonological development [60,62]. As part of this development, increased weight is allocated to acoustic–phonetic cues that are useful in processing their own native language, while the weight of less useful cues is decreased.

Note that Mandarin has word-level stress while Cantonese does not. Although it is widely accepted that Mandarin is a tonal language with lexical tone acting as the dominant suprasegmental cue, stress can also be perceived when a full syllable occurs next to a light syllable [65]. For example, the disyllabic word 东西 means “east and west” with a stressed–stressed pattern ['tʊη'ɕī], while meaning “stuff” with a stressed–unstressed pattern ['tʊηɕi] [28,65]. Shen (1993) examined the necessary acoustic cues underlying the perception of Mandarin stress and found that compared to pitch and intensity, duration plays a dominant role: namely, the duration of the unstressed syllable is dramatically reduced compared to the stressed one [66]. When all three aforementioned acoustic cues (pitch, duration, and intensity) varied simultaneously in pre-attentive English lexical stress perception, Mandarin speakers weighed the duration cue more heavily while Cantonese speakers relied more on pitch information [67]. However, the existence of word-level stress in Mandarin does not imply that a transfer from lexical tone to lexical stress could occur at a phonological level, as the dominant cue involved in Mandarin stress is duration, while in English stress it is vowel quality [28].

## 5. Conclusions

Our study has demonstrated that neural evidence exists to support theories of prosodic transfer from L1 lexical tone to L2 lexical stress. Furthermore, it has shown that both Mandarin and Cantonese speakers rely on pitch cue to perceive English lexical stress and recognize English spoken words. Our results indicate that Cantonese learners rely more on pitch cue than Mandarin learners do, which we attribute to the influence of native language-specific cue weighting. These results are fully in line with cue-weighting theory and provide a straightforward indication of how a speaker’s native language can influence the processing of their second language.

## Figures and Tables

**Table 1:** Mean length, familiarities, word frequency, and standard deviations across conditions in Experiment 1.

	Trochaic Word		Iambic Word	
	*Mean*	*SD*	*Mean*	*SD*
Length	6.26	0.56	6.40	0.88
Familiarity	6.65	0.36	6.50	0.54
Lg10 WF	3.07	0.47	3.00	0.45
Lg10 CD	2.84	0.39	2.79	0.39

**Table 2:** Mean accuracy rates (response times) and standard deviations for the conditions in Experiment 1.

	Mandarin Group		Cantonese Group	
	*Mean*	*SD*	*Mean*	*SD*
Correct word				
Trochaic pitch	0.82 (1265.17)	0.12 (147.34)	0.92 (1142.16)	0.11 (135.48)
Iambic pitch	0.75 (1306.40)	0.10 (119.26)	0.82 (1142.39)	0.13 (135.79)
Incorrect word				
Trochaic pitch	0.68 (1272.36)	0.17 (125.75)	0.75 (1157.09)	0.11 (104.04)
Iambic pitch	0.33 (1337.31)	0.21 (143.43)	0.45 (1218.68)	0.15 (124.78)

**Table 3:** Mean length, familiarities, word frequency, and standard deviations for the two lists of words in Experiment 2.

	Trochaic Word				Iambic Word			
	Target		Filter		Target		Filter	
	*Mean*	*SD*	*Mean*	*SD*	*Mean*	*SD*	*Mean*	*SD*
Length	6.15	1.09	6.31	0.96	6.70	0.80	6.51	0.81
Familiarity	5.40	1.67	5.08	1.44	4.91	1.69	4.74	1.48
Lg10 WF	2.37	0.79	2.39	0.62	2.18	0.59	2.38	0.63
Lg10 CD	2.16	0.75	2.25	0.56	2.02	0.57	2.24	0.59

**Table 4:** Mean accuracy rates (response times) and standard deviations for the conditions in Experiment 2.

	Mandarin Group		Cantonese Group	
	*Mean*	*SD*	*Mean*	*SD*
Segmental_M				
Pitch_M	0.80 (733.24)	0.11 (101.36)	0.81 (624.12)	0.12 (80.27)
Pitch_Mis	0.80 (731.36)	0.15 (104.11)	0.80 (629.06)	0.11 (77.95)
Segmental_Mis				
Pitch_M	0.58 (835.92)	0.22 (85.20)	0.56 (681.80)	0.23 (79.58)
Pitch_Mis	0.60 (819.99)	0.22 (77.76)	0.59 (681.94)	0.27 (79.81)

## Data Availability

The study data will be available from the corresponding author upon reasonable request.
